# Test and Treat TB: a pilot trial of GeneXpert MTB/RIF screening on a mobile HIV testing unit in South Africa

**DOI:** 10.1186/s12879-019-3738-4

**Published:** 2019-02-04

**Authors:** Ingrid V. Bassett, Leah S. Forman, Sabina Govere, Hilary Thulare, Simone C. Frank, Bright Mhlongo, Elena Losina

**Affiliations:** 1 Division of Infectious Disease, Massachusetts General Hospital, Boston, MA USA; 2 Medical Practice Evaluation Center, Department of Medicine, Massachusetts General Hospital, 100 Cambridge Street, 16th Floor, Boston, MA 02114 USA; 3 Harvard University Center for AIDS Research (CFAR), Boston, MA USA; 4 Harvard Medical School, Boston, MA USA; 5 Data Coordinating Center, Boston University School of Public Health, Boston, MA USA; 6 AIDS Healthcare Foundation, Durban, South Africa; 7 Division of General Internal Medicine, Massachusetts General Hospital, Boston, MA USA; 8 Department of Biostatistics, Boston University School of Public Health, Boston, MA USA; 9 Division of Rheumatology, Department of Medicine, and Department of Orthopedic Surgery, Brigham and Women’s Hospital, Boston, MA USA

**Keywords:** Tuberculosis, Test & Treat, GeneXpert MTB/RIF, Community-based screening

## Abstract

**Background:**

Community-based GeneXpert MTB/RIF testing may increase detection of prevalent TB in the community and improve rates of TB treatment completion.

**Methods:**

We conducted a pilot randomized trial to evaluate the impact of GeneXpert screening on a mobile HIV testing unit. Adults (≥18y) underwent rapid HIV testing and TB symptom screening and were randomized to usual mobile unit care (providing sputum on the mobile unit sent out for GeneXpert testing) or the “Test & Treat TB” intervention with immediate GeneXpert testing. Symptomatic participants in usual care produced sputum that was sent for hospital-based GeneXpert testing; participants were contacted ~ 7 days later with results. In the “Test & Treat TB” intervention, HIV-infected or HIV-uninfected/TB symptomatic participants underwent GeneXpert testing on the mobile unit. GeneXpert+ participants received expedited TB treatment initiation, monthly SMS reminders and non-cash incentives. We assessed 6-month TB treatment outcomes.

**Results:**

4815 were eligible and enrolled; median age was 27 years (IQR 22 to 35). TB symptoms included cough (5%), weight loss (4%), night sweats (4%), and fever (3%). 42% of eligible participants produced sputum (intervention: 56%; usual care: 26%). Seven participants tested GeneXpert+, six in the intervention (3%, 95% CI 1%, 5%) and one in usual care (1%, 95% CI 0%, 6%). 5 of 6 intervention participants completed TB treatment; the GeneXpert+ participant in usual care did not.

**Conclusion:**

GeneXpert MTB/RIF screening on a mobile HIV testing unit is feasible. Yield for GeneXpert+ TB was low, however, the “Test & Treat TB” strategy led to high rates of TB treatment completion.

**Trial registration:**

This study was registered on November 21, 2014 at ClinicalTrials.gov (NCT02298309).

## Background

The dual epidemics of HIV and TB continue unabated in many urban areas in resource-limited settings, and TB remains a leading cause of death among HIV-infected people in sub-Saharan Africa [1–3]. Despite substantial investment in healthcare facility-based diagnosis and treatment of TB, only a fraction of HIV-infected people and others at risk for TB in South Africa are screened for TB and complete TB treatment; therefore TB prevention and care at the community level remains poor [3]. Through early detection, active, mobile, community-based TB case finding may add substantially to facility-based efforts and may improve both individual outcomes and TB control at the population level [4].

GeneXpert MTB/RIF (Cepheid GeneXpert System, Sunnyvale, CA), a rapid, automated molecular diagnostic tool, currently deployed largely within centralized provincial hospitals in South Africa, has great potential for intensified case finding at the community level among people not accessing clinic-based services [5–7]. Building on the theoretical foundation of the Test and Treat approach for HIV [8–10], we conducted a pilot randomized controlled trial to evaluate the yield of GeneXpert MTB/RIF on a mobile HIV testing unit operating in community venues in Umlazi Township, Durban. We assessed integrating GeneXpert MTB/RIF and HIV screening, maximizing the impact of mobile units, which typically refer patients with suspected TB to local clinics for evaluation and treatment [11, 12]. Our objective was to establish the feasibility of a program that: 1) uses GeneXpert screening on a mobile testing unit, and 2) shortens time to TB treatment initiation compared to clinic-based referral for patients to be evaluated for TB, with the goal of increasing TB treatment completion rates.

## Methods

### Setting and participants

We performed a randomized trial to evaluate the yield of GeneXpert MTB/RIF on a mobile HIV testing unit operating in community venues in Umlazi Township (ClinicalTrials.gov NCT02298309). The eThekwini District, which encompasses Umlazi, has a TB incidence of 916 per 100,000 and an HIV prevalence of 15% [13, 14]. Participants were enrolled on the iThembalabantu Tester, a nurse-run, counselor-supported mobile HIV screening unit. Affiliated with iThembalabantu Clinic, the unit operates at community venues such as taxi stands, petrol stations, and markets, where staff set up tents for HIV testing. All adults (≥18y), English- or Zulu-speaking, were eligible if they were: voluntarily undergoing HIV testing and not known previously to be HIV-infected, able and willing to provide informed consent, willing to share test results with study staff, willing to follow-up at one of the prespecified study clinics, had access to a mobile phone, and were not known to be pregnant. Women known to be pregnant were excluded from the study due to more aggressive referral, HIV and TB treatment monitoring, and follow-up pathways within the South African health system for them. All HIV-infected participants were eligible for TB screening, as well as HIV-uninfected participants endorsing any TB symptoms.

The study was approved by the University of KwaZulu-Natal Biomedical Research Ethics Committee and the Partners Institutional Review Board.

### Randomization

For feasibility reasons, randomization occurred by day, and was stratified by mobile sites, in blocks of variable size. All patients seen at the mobile unit on a given day were enrolled in the same strategy to simplify logistical considerations and minimize the risk of participants interacting with participants in another study strategy. Randomization assignments were generated and accessed electronically each morning to prevent contamination.

### Enrollment procedures

Clients waiting for HIV testing were approached by a bilingual research assistant (English/Zulu) to assess interest in study participation. Eligible and consenting participants were asked a short set of screening questions. A Tester staff member administered a brief questionnaire regarding prior HIV testing history, TB diagnosis and treatment history, and TB symptoms. Participants were asked to provide their mobile phone number and contact phone number of a friend/family member. To assist with follow-up, we recorded the name of the clinic patients anticipated receiving care, in case of a positive HIV and/or GeneXpert MTB/RIF test. Participants then underwent rapid HIV testing as per South African protocol. HIV-infected participants were offered a point-of-care CD4 count (Alere PIMA™ Analyzer, Waltham, MA). HIV-infected participants were given a clinic referral letter detailing next steps for obtaining HIV treatment.

### Test and Treat TB intervention

Participants in the Test and Treat TB intervention were asked to produce a sputum sample by a dedicated research nurse on the mobile unit. Participants in the intervention screened with GeneXpert received their TB test results by SMS in English or Zulu, based on preference. Asymptomatic participants with a negative GeneXpert MTB/RIF received results by SMS and were not asked to return to the Tester. Symptomatic participants with a negative GeneXpert MTB/RIF test were instructed to report to a local clinic for a sputum culture [15]. Participants with a positive GeneXpert MTB/RIF test were asked to return to the Tester.

GeneXpert-positive participants, regardless of HIV status, were provided: 1) a TB treatment starter pack, a three-week supply of a weight-based, fixed-dose combination of rifampin, isoniazid, pyrazinamide, and ethambutol, as per South African guidelines, dispensed at the mobile tester, and referral to a local clinic with a copy of their GeneXpert MTB/RIF test result to continue treatment [16], 2) SMS reminders at the end of the starter pack and monthly for the duration of TB treatment and 3) three cashless incentives (mobile phone minutes) for a) returning to the mobile unit for positive test results, b) linkage to TB care at a participating clinic (within 3 weeks), and c) TB treatment completion.

### Usual care

Participants with TB symptoms in usual care were provided verbal instructions by mobile tester nurses to produce a spot sputum sample on the mobile unit, which was driven daily to the provincial hospital for GeneXpert MTB/RIF testing. Participants were phoned by the clinic-based TB nurse ~ 7 days later with results and treatment referral, either to iThembalabantu Clinic or to the participant’s selected clinic of choice. Symptomatic participants who could not produce a sputum, regardless of HIV status, were referred to a local clinic.

### TB sputum collection and sample processing

All nurses were trained in standard local infection control practices, including N95 masks for personnel and sputum collection in a dedicated outdoor area away from waiting areas. To increase likelihood of obtaining sputum samples, nurses provided verbal instructions, encouraged participants to use a nebulizer and offered a private space for participates to expectorate a spot sputum specimen. Staff from the local Cepheid office assisted with GeneXpert technical support and staff training. The GeneXpert device was plugged into a generator and equipped with a back-up uninterrupted power source. A trained research nurse prepared sputum specimens with results available on a printable readout in ~ 100 min.

### Outcome measures

#### Primary outcomes

The primary outcome was the proportion of GeneXpert-positive individuals who completed six months in TB care. For participants in usual care, TB diagnosis was confirmed through GeneXpert results from the National Health Laboratory Service. For participants in the intervention, TB was defined as a positive GeneXpert MTB/RIF on the mobile unit.

#### Secondary outcomes

Secondary outcomes included: 1) proportion of mobile testers screened for TB; 2) proportion of eligible participants able to produce sputum samples; 3) prevalence of TB among those tested; 4) prevalence of rifampin resistance among those testing positive for TB; 5) distribution of TB symptoms; 6) return for TB test result; 7) linkage to TB care (first visit to a local clinic) and 8) ART initiation for those HIV co-infected. Participants with rifampin resistance were referred to King George V Hospital for treatment. We documented the number of unique sites visited by the Tester, as well as the frequency of mobile unit visits by site designation (e.g. mall, transit area) and the proportion of TB cases identified in each category.

#### Outcome assessments

We assessed linkage to TB for all HIV-infected or HIV-uninfected participants with a positive GeneXpert MTB/RIF or with TB symptoms referred to a local clinic. Three weeks after enrollment, we contacted clinics to ascertain attendance at the first TB and/or HIV visit. We also contacted clinics at six months to assess retention in care. Nine months after enrollment we accessed the National Tuberculosis Control Programme database to confirm clinic-reported TB treatment outcomes.

### Sample size and power

We estimated 15% HIV prevalence on the mobile tester [17], and ~ 7% TB prevalence among individuals newly diagnosed with HIV [18]. Assuming 700 people entered the Tester/month, we estimated ~ 105 (0.15*700) would be HIV-infected and screened for TB/month. At a prevalence of 7%, we expected about 8 patients with TB/month. We based our TB treatment completion rate in usual care on the 2012 WHO Global Tuberculosis Report for TB. Given the limited sample in a pilot, we focused on precision of the estimated treatment benefit that “Test and Treat TB” would provide. Assuming a TB treatment completion rate of 53% in usual care, the 95% confidence limits for various estimated improvements in the rate were: 10% (− 9.3, 29.3%), 15% (− 4.0, 34.0%) and 20% (1.4, 38.6%).

### Analysis plan

We used descriptive statistics and bivariate analyses to examine the success of randomization in balancing variables that may affect outcome (e.g. age, sex). The primary goal was to establish the effect size, expressed as a relative difference in TB treatment completion rates, between the Test and Treat TB intervention and usual care; however, due to lower than anticipated case finding, we switched focus from primary to secondary outcomes. Using an intent-to-treat approach, participants were analyzed in the group they were assigned. In an additional analysis focusing on individuals in either arm eligible to give a sputum sample, we used Poisson regression to examine the effect of sex, age, number and type of TB symptoms, and HIV status on ability to produce a sputum sample.

## Results

### Enrollment

**Fig. 1:**
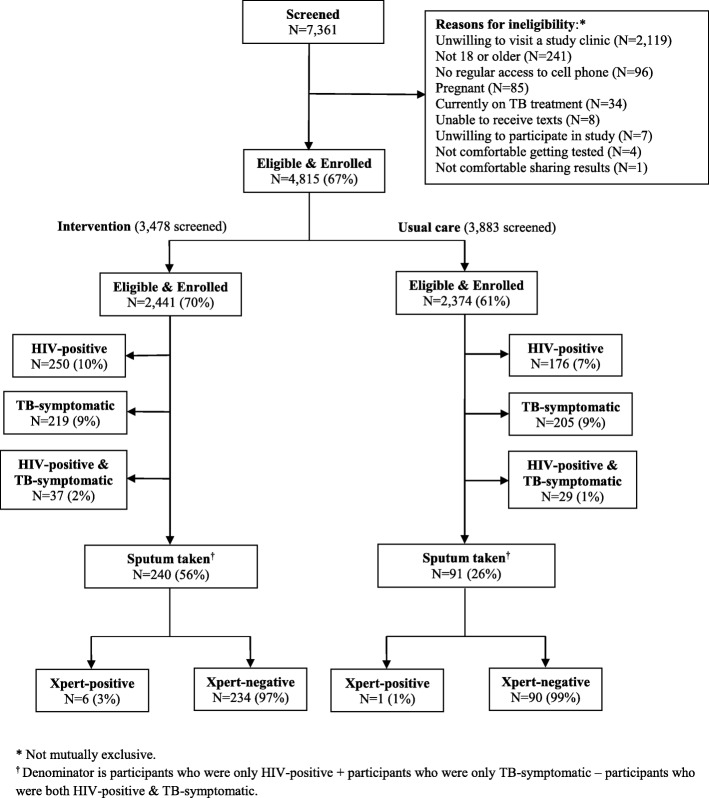
Participant Flow. Of 7361 individuals (intervention: 3478; usual care: 3883) screened over 20 months, 4815 (intervention: 2441; usual care: 2374) were eligible and enrolled. The main reason for ineligibility was unwillingness to visit one of the follow-up clinics (2119, 88%). Other reasons for ineligibility included: age < 18 years (241, 10%); no regular access to a cell phone (96, 4%); pregnant (85, 4%); currently on TB treatment (34, 1%). Being unable to receive texts, unwilling to participate in the study, not comfortable getting tested, or not comfortable sharing results comprised < 1% of ineligibility. In the intervention arm, 250 (10%) participants were HIV-positive, 219 (9%) were TB-symptomatic, and 37 (2%) were both HIV-positive and TB symptomatic. Of those eligible to give a sputum sample, 240 (56%) successfully produced sputum and 6 (3%) tested GeneXpert-positive. In the usual care arm, 176 (7%) participants were HIV-positive, 205 (9%) were TB-symptomatic, and 29 (1%) were both HIV-positive and TB symptomatic. Of those eligible to give a sputum sample, 91 (26%) successfully produced sputum and 1 (1%) tested GeneXpert-positive

Enrollment was from April 2015–November 2017. Of 7361 screened (intervention: 3478; usual care: 3883), 4815 (intervention: 2441; usual care: 2374) were eligible and enrolled (Fig. 1). The main reason for ineligibility was unwillingness to visit a prespecified follow-up clinic (2119, 86%). Other reasons for ineligibility included: age < 18 years (241, 10%); no regular cell phone (96, 4%); pregnancy (85, 3%); current TB treatment (34, 1%). These categories were not mutually exclusive. Among enrollees, median age was 27 years (IQR 22 to 35), 51% were male, and 95% reported prior HIV testing (Table 1). HIV prevalence was estimated at 8.8% (95% CI 8.2, 9.8% (*n* = 426)). Of note, this estimate was ~ 1/2 of anticipated prevalence, likely due to widespread HIV testing, with 95% of participants reporting prior HIV testing.

**Table 1:** Baseline characteristics of enrolled study participants

	Intervention (*n* = 2441)	Usual care (*n* = 2374)	Overall (*n* = 4815)
Age mean years (SD)	31 (13)	31 (13)	31 (13)
Sex			
Female n, (%)	1206 (51)	1109 (48)	2315 (49)
Male n, (%)	1181 (49)	1200 (52)	2381 (51)
Prior HIV testing			
Yes n, (%)	2173 (95)	2112 (95)	4285 (95)
No n, (%)	104 (5)	121 (5)	224 (5)
HIV status			
Positive n, (%)	250 (9)	176 (7)	426 (9)
CD4 mean (SD)	435 (234)	498 (345)	440 (246)
CD4 median (IQR)	419 (264, 592)	413 (205, 769)	419 (259, 600)
TB symptoms			
Cough n, (%)	129 (5)	99 (4)	228 (5)
Weight loss n, (%)	84 (4)	89 (4)	173 (4)
Night sweats n, (%)	102 (4)	89 (4)	191 (4)
Fever n, (%)	82 (3)	56 (2)	138 (3)
TB prevalence n, (%)	6 (0.2)	1 (0.0)	7 (0.1)

*SD* Standard deviation

*IQR* Interquartile range

### Mobile tester

The mobile tester visited 379 unique sites (intervention days: 192 sites; usual care days: 187 sites). The site visits per designation were: transit area: 34%; residential community area: 27%; mall/community store: 22%; open sports ground community area: 6%; home: 5%; university/school: 4%; other (playground, community hall, unknown): 2%. Site distribution did not differ between study arms.

### TB screening

TB symptoms included cough (5%), weight loss (4%), night sweats (4%), and fever (3%). Distribution of TB symptoms was similar across study arms. Among HIV-positive participants in the intervention, mean CD4 count was 435 cells/μl. Overall, seven participants tested GeneXpert-positive; six of these were in the intervention (3%, 95% CI 1%, 5%) and one in usual care (1%, 95% CI 0%, 6%). All GeneXpert-positive participants were identified in residential community and transit areas.

### Factors influencing obtaining sputum

Overall, 42% of eligible participants produced sputum samples (intervention: 56%; usual care: 26%). Among intervention participants, only 41% exhibiting no TB symptoms successfully produced sputum compared to 48% in those with one symptom, 71% with two, 72% with three and 89% with four. HIV-positive participants without TB symptoms were twice as likely not to provide sputum compared to HIV-positive participants with symptoms (RR 2.01, 95% CI 1.05–3.85) (Table 2). Among those with TB symptoms, HIV-negative individuals were 64% more likely not to provide sputum compared to HIV-positive (RR 1.64, 95% CI 0.85–3.19). Those tested at commercial areas were 40% more likely not to provide sputum (RR 1.40, 95% CI 1.00–1.95 compared to community areas/home). Younger age was independently associated with higher likelihood of not providing sputum (RR 1.15 CI 1.01–1.31, per decade).

**Table 2:** Factors affecting the likelihood of not providing a sputum sample among participants eligible for sputum collection, adjusted Poisson model

Factor	Adjusted iRR (95% CI)	*p*-value
Clinical characteristics		
HIV-positive & no TB symptoms vs. HIV-positive & TB-symptomatic	2.01 (1.05, 3.85)	0.0344
HIV-negative & TB-symptomatic vs. HIV-positive & TB-symptomatic	1.64 (0.85, 3.19)	0.1433
Mobile testing location		
Mall/Other vs. Community area/Home	1.40 (1.00, 1.95)	0.0477
Transit area vs. Community area/Home	1.11 (0.79, 1.57)	0.5492
Cohort characteristics		
Age, per decade	1.15 (1.01, 1.31)	0.0380

*iRR* incidence rate ratio

### Outcomes for TB-positive participants

Of seven participants who tested GeneXpert-positive, median age was 37 years (IQR 29 to 50), 29% were male, and 71% (5/7) were HIV-positive. Three of seven (43%) GeneXpert-positive participants had TB symptoms. Of six participants who tested GeneXpert-positive in the intervention, three (50%, 95% CI 36%, 88%) linked to the TB clinic within 3 weeks. All six in the intervention linked to care within 6 months, and 5 of 6 (83% 95% CI 36%, 100%) completed treatment. The single GeneXpert-positive participant in usual care did not link to TB care. Rifampin resistance was detected in one of six GeneXpert-positive intervention participants. Four of five (80%) GeneXpert-positive, HIV-positive participants initiated ART; mean CD4 count for the TB-positive, HIV-infected enrollees in the intervention was 246 cells/μl.

## Discussion

We conducted a pilot randomized trial to evaluate the yield of GeneXpert MTB/RIF on a mobile HIV testing unit operating in community venues in Umlazi Township, Durban. Overall, only 42% of eligible participants could produce sputum (intervention: 56%; usual care: 26%). Seven participants tested GeneXpert-positive; six of these participants were in the intervention and one was in usual care. Of the seven participants who tested GeneXpert-positive, 71% were HIV-positive and 43% were TB symptomatic at enrollment. In the intervention arm, 5 of 6 participants completed TB treatment by 6 months; the sole GeneXpert-positive participant in usual care did not link to TB care.

While this trial demonstrates that adding TB screening to mobile HIV testing unit activities in the community using GeneXpert MTB/RIF is feasible, overall TB yield was low. The low observed prevalence was consistent with another recent South African TB screening study performed in the context of mobile, community-based HIV testing, in which only a third of participants with at least one TB symptom underwent sputum testing and TB prevalence was 3%. [19]. In our study, it was difficult for many participants to produce sputum on the mobile unit even with a nebulizer, and sputum collection was not equal across arms. Without a dedicated study nurse on the mobile tester during usual care days, fewer participants produced sputum in the usual care arm, and we may have thus underestimated the TB prevalence in our population. Although specimen production was better as the number of TB symptoms increased, implementing generalized, community-based pulmonary TB screening may be more effective with enhanced sputum induction techniques. Additionally, TB yield may have been lower than expected because the mobile tester reached an apparently healthier population with lower HIV and TB prevalence than our original estimates. Mean CD4 count for HIV-infected individuals presenting to the mobile unit was high (440 cells/μl) compared to clinic-based facilities [20, 21]. This healthier mobile testing population could also be indicative of the success of community-based HIV testing led by iThembalabantu Clinic, which has been offering mobile HIV testing in Umlazi since 2008, and which may account for 95% of participants reporting prior HIV testing.

This pilot trial was unique in its adaptation and application of the Test and Treat HIV theoretical framework to an integrated, community-based TB screening and treatment program. By deploying GeneXpert MTB/RIF outside of a central site and offering immediate TB treatment initiation outside of the clinic, we attempted to expedite TB case finding in the community. While we found only a small number of TB cases in the intervention arm, TB treatment completion rates in the “Test & Treat TB” strategy were better than completion rates for TB cases diagnosed at clinic level in South Africa (5 of 6, 83%, 95% CI 36%, 100%) [16, 22]. Participants were enrolled prior to their HIV and/or TB test to reduce differential acceptance rates by results of HIV and TB testing, facilitating a representative sample of newly diagnosed individuals. The substantial number of unique mobile testing sites allowed for enrollment of participants throughout the community and was representative of the population of mobile testers across Umlazi.

This study had several limitations. The most common reason for ineligibility was unwillingness to visit one of the prespecified study clinics. Although we added follow-up clinic locations over the study period, nearly 30% of individuals screened were unwilling to visit a participating clinic. This may have been due to the largely ambulatory population attracted by the mobile tester, who were not necessarily testing close to their residence. Because of this, we were not able identify TB cases within the group of individuals who were screened, but not enrolled. Despite block randomization by day, a significantly greater proportion of individuals were eligible and enrolled in the intervention arm of the study; it is difficult to know why this occurred, but perhaps mobile tester clients were made aware of the presence of the GeneXpert on intervention days and were more likely to enroll in the study. For GeneXpert-positive study participants in both study arms, we reviewed their medical record at the prespecified follow up clinic, however, participants could enter care at other clinics offering TB and HIV care. We addressed this by also accessing the Department of Health TB Control Programme and National Health Laboratory Services, which are not clinic-specific. Additionally, incorporating GeneXpert MTB/RIF testing in the community posed logistical challenges; as seen in other studies in KwaZulu-Natal, many eligible participants were not able to produce sputum on the mobile unit, which may have restricted the yield of new TB cases [23]. Participants may have also felt inhibited to produce sputum in community venues. For those who were able to produce sputum, false negative diagnoses may have resulted from the limited sensitivity of the current GeneXpert MTB/RIF platform for a single sputum specimen; sensitivity may improve in the future with next generation assays for TB screening. Sputum culture may also increase sensitivity compared to GeneXpert on a single sputum specimen, which had a sensitivity of 86% in South Africa in a prior study [24], however, our protocol was consistent with the current South African guidelines which recommends a single GeneXpert as the initial step for screening people with TB symptoms [15].

## Conclusion

Despite the continued development of facility-based programs in sub-Saharan Africa for diagnosis and treatment of TB, little has been accomplished to improve prevention and care for TB at the community level. Screening for TB in the community using the rapid, diagnostic tool GeneXpert MTB/RIF is feasible and may assist in expanding access to TB testing and treatment completion. The impact of a community-based Test and Treat TB program could be even greater with enhanced sputum induction techniques and more sensitive screening tests. This trial has the potential to inform physicians, governments, and policy makers on how to maximize the benefits of community screening through timely and integrated mobile HIV/TB diagnosis and linkage to care.

## Abbreviations

ART: Antiretroviral therapy
CI: Confidence interval
HIV: Human immunodeficiency virus
IQR: Interquartile range
iRR: Incidence rate ratio.
MTB: *Mycobacterium tuberculosis*
RIF: Resistance to rifampin
RR: Relative risk
SD: Standard deviation
SMS: Short message service
TB: Tuberculosis
WHO: World Health Organization
